# Edge-Computing Video Analytics for Real-Time Traffic Monitoring in a Smart City

**DOI:** 10.3390/s19092048

**Published:** 2019-05-02

**Authors:** Johan Barthélemy, Nicolas Verstaevel, Hugh Forehead, Pascal Perez

**Affiliations:** SMART Infrastructure Facility, University of Wollongong, Wollongong, NSW 2522, Australia; nicolasv@uow.edu.au (N.V.); hughf@uow.edu.au (H.F.); pascal@uow.edu.au (P.P.)

**Keywords:** edge-computing, IoT, smart city, video analytic, traffic monitoring, CCTV

## Abstract

The increasing development of urban centers brings serious challenges for traffic management. In this paper, we introduce a smart visual sensor, developed for a pilot project taking place in the Australian city of Liverpool (NSW). The project’s aim was to design and evaluate an edge-computing device using computer vision and deep neural networks to track in real-time multi-modal transportation while ensuring citizens’ privacy. The performance of the sensor was evaluated on a town center dataset. We also introduce the interoperable *Agnosticity* framework designed to collect, store and access data from multiple sensors, with results from two real-world experiments.

## 1. Introduction

With massive increases in the world’s population and more than 60% of the world population projected to live in urban areas, cities face serious urban planning challenges [1]. Not only do they face rapidly growing population, but they also have to deal with social and sustainability challenges. To better cope with changes, cities need long-term approaches leading to sustainability [2].

Rethinking cities to not only efficiently manage their current situation and population, but also their future growth is exactly the main motivation behind the concept of smart cities. While there is no consensual definition of what a smart city is [3], it commonly involves the usage of Information and Communication Technologies (ICT) to design tools which should respond to people’s needs through sustainable solutions for social and economic challenges.

Bibri and Krogstie [4,5] proposed an interdisciplinary literature review of smart and sustainable cities and pointed out the interest of a new generation of urban planning tools for improving mobility and accessibility. A smart city is then a significant tool for municipalities which can reduce the spending and perform real-time monitoring of their transportation, energy and utilities networks [6].

Currently, many Australian cities are rapidly developing their existing CCTV network. These large networks represents a major cost for the councils in terms of maintenance, but are only used for investigating incidents and monitor anti-social behaviors in public places [7]. Due to stringent privacy regulations, only the police and a few accredited operators are allowed to view the live or recorded video feed. This results in the expensive collection of a vast amount of rich data that have been unused thus far. This paper presents a new sensor, based on the edge-computing paradigm, for real-time traffic monitory leveraging the existing CCTV network data to address the issues of the network cost by adding new usages while respecting the privacy regulations.

The remainder of this paper is organized as follows. Section 2 introduces the scope of the project, its requirements and methodology, which lead to the development of the sensor introduced in Section 3. Section 4 then focuses on the framework designed to collect, store and access the data. The performances of the sensor are assessed in Section 5. We next present in Section 3 two applications of the sensor. Concluding remarks and future work are discussed in Section 7.

## 2. The Liverpool Smart Pedestrians Project

The Liverpool Smart Pedestrians project was funded under the Australian Government Smart Cities and Suburbs Program for a duration of one year starting from February 2018. It was a collaboration between the Liverpool city council and the University of Wollongong. The project aimed to design innovative solutions for the collection of data in a non-intrusive way to help inform urban planning in Liverpool, a suburb of Sydney in New South Wales, Australia. It is located in the Greater Western Sydney, 32 km southwest of the Sydney central business district and has an estimated population of 27,084 citizens. The city is growing rapidly, with more housing, offices and educational facilities. The council’s redevelopment of its CBD is expected to bring in 30,000 additional pedestrian per day. All of this makes the city a good area for experiments monitoring the effect of this redevelopment on the traffic.

### 2.1. Methodology and Objectives

At the very beginning of the project, two community workshops were conducted: an afternoon session with 25 participants and an evening session with 10 participants. The results of the community workshops and the feedback from city urban planners highlighted the need for a sensor monitoring the traffic with the following requirements:

- Multi-modal detection and tracking: The sensors need to be able to detect and track pedestrians, vehicles and cyclists.
- Privacy compliant: As sensors are going to be deployed over a city, the sensors should be privacy compliant, meaning that no personal data should be stored or exchanged.
- Leveraging existing infrastructures: As cities already make huge investments on CCTV systems [8], the solution should take advantage of the already existing infrastructures in terms of networks and cameras. Retrofitting the existing CCTV network to collect more data has been identified as a major innovation.
- Scalability and interoperability: New sensors can be added at any time, regardless their technologies, meaning the sensor of network can be easily expanded and capture new type of data.

The work detailed in this paper thus proposes a contribution to the field of traffic monitoring using video analytics.

### 2.2. Related Work

The first stage for monitoring and modelling traffic in a road network is collecting traffic counts. Inductive loop detectors, pneumatic road tubes, and temporary manual counts have been the primary methods for collecting such traffic data [9]. The development of automatic sensing technologies, to replace manual counting, has allowed a higher frequency rate as well as the permanent monitoring of the traffic counts [10]. Other classic traffic counter devices include piezo-electric sensors and radar-based off-roads sensors [11,12]. While initially being designed for vehicular traffic, most of them can also be adapted to count bicycles and pedestrians [13].

The advent of the Internet of Things (IoT) has enabled the development of new traffic and pedestrian counting technologies relying on the usage of multiple sensors deployed over a network. The technology can track road users through their smartphones, NFC, GPS and connected traffic counters [14]. Some of those sensors can be deployed in a meshed configuration to perform traffic counting without having to make large investment in a new infrastructure [15,16]. This new generation of connected and distributed sensors allows the collection of a greater amount of data at a very fine level. This offers two main benefits: a better representation of the traffic and the emergence of data-driven traffic models [17].

Romero et al. [18] proposed a literature review of various sensing methods used for traffic detection and surveillance. They compared various technologies, such as inductive loop, magnetic induction or video image processing, pointing out some of the advantages and drawbacks. As a conclusion, they highlighted that the sensors based on video cameras offer a relatively low installation cost with little traffic disruption during maintenance, whereas other methods such as inductive loop, RADAR and microwave detectors suffer from serious drawbacks.

With the drastic reduction in the cost of electronic components, and recent advances in machine learning and images processing, it is now possible to develop at relatively low cost, edge computing solutions to monitor traffic. For example, Gupta et al. [19] designed low-cost hardware using Wi-Fi strength as a signal to monitor traffic. The passage of a car between a transmitter and a receiver produces a variation in signal strength that can be measured to count vehicles flows. However, this approach still needs new infrastructures.

Another approach is to rely on already existing infrastructures to perform real-time monitoring. Indeed, as cities have been massively investing in CCTV networks [8], retrofitting the already existing CCTV infrastructure to transform classical CCTV into smart CCTV becomes a promising approach to real-time monitoring of traffic. Consequently, more and more research is being done using video analytics on CCTV footage. For example, Kim et al. [20] used CCTV in an urban traffic information system to determine traffic speed and volume and combine this information with on board wireless equipment to estimate travel speed.

Ganansia et al. [21] used face recognition algorithms to study the spatiotemporal behavior of people in a railway station. They tested their approach in train and subway stations in Paris and Turin. While they were able to extract daily profiles, the need to set-up and optimize algorithms for each specific viewpoint is an obstacle for large deployment, mainly due to poor conditions of image acquisition such as low resolution, lighting conditions, combined with very dense crowds.

The recent advances in neural network architectures now allows the training algorithms on large datasets to reduce the need for manual tuning. For example, Dimou et al. [22] used a Faster R-CNN neural network architecture [23] to track objects in CCTV footage in order to take into account blur and Pan–tilt–zoom features of CCTVs. They showed that use of heterogeneous data and data augmentation with motion blur during the training phase can improve the performance of the detector.

Peppa et al. [24] evaluated the performances of the Single Shot MultiBox Detector MobileNet neural network, and two versions of Faster R-CNN trained on the KITTI [25] and the COCO [26] datasets on CCTV footage captured on different weather conditions. Their results show that a fine-tuned MobileNet model can achieve 98.2% precision, 58.5% recall and 73.4% harmonic mean, making it a potential candidate for a real time traffic monitoring application with big data due to its fast performance. A fine-tuned Faster R-CNN model provides a better harmonic mean (80.4%), better recall (68.8%) and more accurate estimations of car units, and could be used for traffic analysis applications that demand higher accuracy than speed.

Acharya et al. [27] used a Faster R-CNN neural network architecture for object detection and tracking. They evaluated their algorithm on the town center dataset [28]. The tracking algorithm is able to track pedestrians with 71.13% accuracy at a rate of four frames per seconds. To improve the performance, they proposed the usage of Kalman filters for overcoming the problem of unpredictable pedestrian movements.

The usage of CCTV footage is not restricted to traffic counting but can also be used for predicting accidents [29], to extract velocity in crowd to increase safety and comfort [30], or can even be used to detect firearms and knives [31].

It can be seen that the usage of neural networks to track mobility in a city by using CCTV footage is a promising approach. However, most of the approaches in the literature performs offline analysis of recorded CCTV footage. Furthermore, to the authors’ knowledge, no approach has proposed to use real-time data analysis as an urban planning tool and has deployed this tool at the scale of a city.

Finally, it is clear that, when using CCTV footage, ensuring privacy is a major issue. As noted by Satyanarayanan et al. [32,33] and Shi et al. [34,35], the edge computing paradigm offers a way to process data at the edge of the network to address concerns such as bandwidth saving, as well as data safety and privacy. Indeed, the privacy of the data is ensured by the processing which denatures the raw data. The resulting data are typically smaller than the original raw data. The design of an edge computing device with the ability to perform real-time analysis of CCTV would then allow not only to collect data but also to ensure privacy as the image feeds would not leave the already existing CCTV network and only denatured data would be produced by the device.

### 2.3. Pilot Project

**Figure 1:**
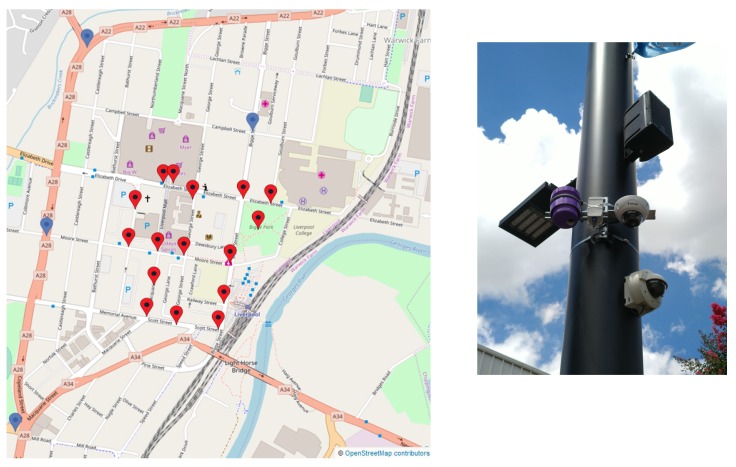
A map of the town center. (**left**) Locations of the 20 visual sensors. It is expected that the live data produced from those location will help urban planners to update the city’s mobility plan. (**right**) Co-location of two CCTV cameras and an air quality (in purple) on a pole.

The project aims to develop and evaluate mobility trackers using CCTV live feeds. The town center of Liverpool was selected as test bed. Twenty sensors will be deployed over the city center to monitor traffic flows. Fifteen of them will use already existing CCTVs while five of them will use mobile CCTVs allowing relocation if needed. Figure 1 displays a map of the town center and the location of the sensors. As part of this pilot project, 20 air quality and noise sensors will be co-located with the mobility trackers to evaluate the impact of the traffic on air quality and noise pollution. An example is shown in Figure 1. However, those sensors are not discussed in this paper.

In the following section, we introduce the design of the sensor to monitor the mobility by detecting and tracking vehicles, people and bicycles.

## 3. An Edge-Computing Device for Traffic Monitoring

This work proposes and evaluates a sensor that meets the requirements highlighted in Section 2.1. The objective is to deploy a fleet of these sensors enabling citywide traffic monitoring in real-time. Firstly, we introduce the sensor functionality and its hardware. Then, we describe and motivate the choice of the software components combining a detection algorithm with a tracking algorithm.

### 3.1. Functionality and Hardware

For the purpose of monitoring the mobility within a network, we have designed a sensor that is able to detect and track objects of interest in a live video feed using video analytics. The most important feature of the sensor is that it follows the edge-computing paradigm, i.e., the video analytics are run directly on the device and only the results of the processing are transmitted. This has two main advantages:

- it lowers the network bandwidth requirement as no raw images is transmitted, but only indicators and meta-data; and
- thanks to the limited amount of information being transmitted, the device is privacy compliant.

The privacy compliance of the device is critical for real world applications and deployment in smart cities. Indeed, the system can be paired with existing CCTV infrastructure while not transmitting the actual video feed captured from the cameras. This lowers the deployment cost of the sensor as no additional camera is needed while allowing uses of the already existing CCTV infrastructure.

The device has the ability to transmit the outputs either over Ethernet or LoRaWAN networks, the latter being a wireless long range, low power network for the Internet of Things [36]. The limited bandwidth and duty cycles available to LoRaWAN devices further justify the use of edge-computing.

**Figure 2:**
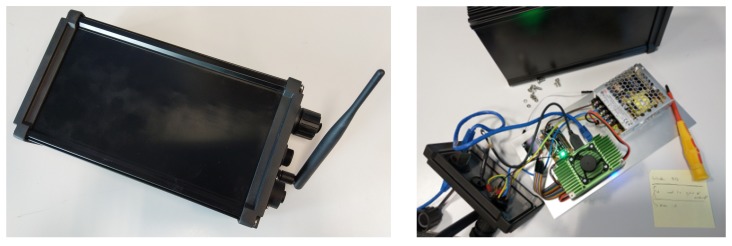
The smart visual sensor, outside and inside.

The prototype, illustrated in Figure 2 has two core components:

- an NVIDIA Jetson TX2, a high performance and power efficient ARM-based embedded computing device with specialized units for accelerating neural network computations used for image processing and running Ubuntu 16.04 LTS; and
- a Pycom LoPy 4 module handling the LoRaWAN communications on the AS923 frequency plan used in Australia. It should be noted that the module is able to transmit on every frequency plan supported by the LoRaWAN protocol.

**Figure 3:**
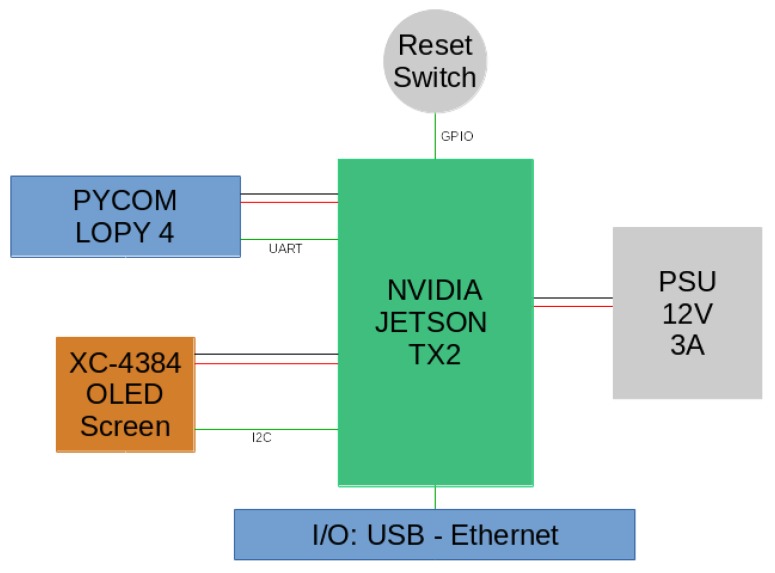
Simplified diagram of the sensor. The core of the sensor is the Jetson TX2 handling the video analytics, the USB and Ethernet connectivity and powering the LoPy 4 and the OLED screen. The Jetson TX2 is in turn powered by the PSU. The LoPy is used for LoRaWAN-based transmission and the screen to display basic information about the sensor’s status.

Their main technical specifications are listed in Appendix A. The sensor is paired with an XC-4384 compact monochrome OLED module from DuinoTECH for displaying the state of the sensor. A 35 W power supply unit is responsible for powering the device. A simplified diagram of the sensor’s components interconnection is shown in Figure 3. Finally, the sensor is packaged inside a waterproof IP67 rated aluminum heat sink case. This case is suited for outdoor environments and is able to dissipate the heat produced by the Jetson TX2.

The sensor performs the following steps iteratively on average 20 times each second:

1. Frame acquisition from an IP camera or an USB webcam.
2. Detecting the objects of interests in the frame.
3. Tracking the objects by matching the detections with the ones in the previous frame.
4. Updating the trajectories of objects already stored in the device database or creating records for the newly detected objects.

**Figure 4:**
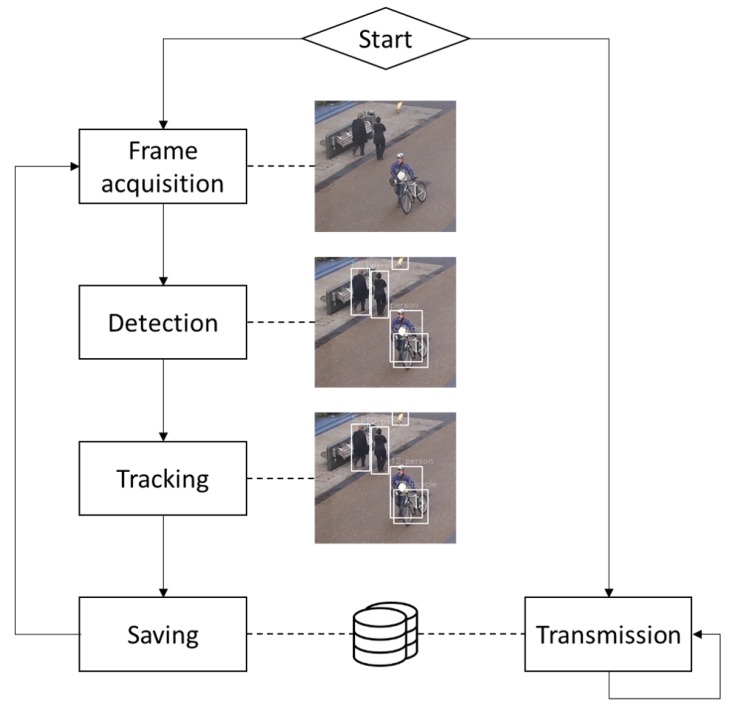
Activity flow chart of the sensor.

In parallel to those tasks, the sensor periodically transmits the results from the video processing to the IoT Core, detailed in Section 4, either via LoRaWAN or Ethernet. The time interval between two successive transmissions can be set by the user, but can not be less than 5 min if LoRaWAN is used, due to the constraints of the protocol. After each transmission, the local database is emptied to prevent the saturation of the local storage (32 Gb). The activity flowchart of the sensor is represented in Figure 4.

**Figure 5:**
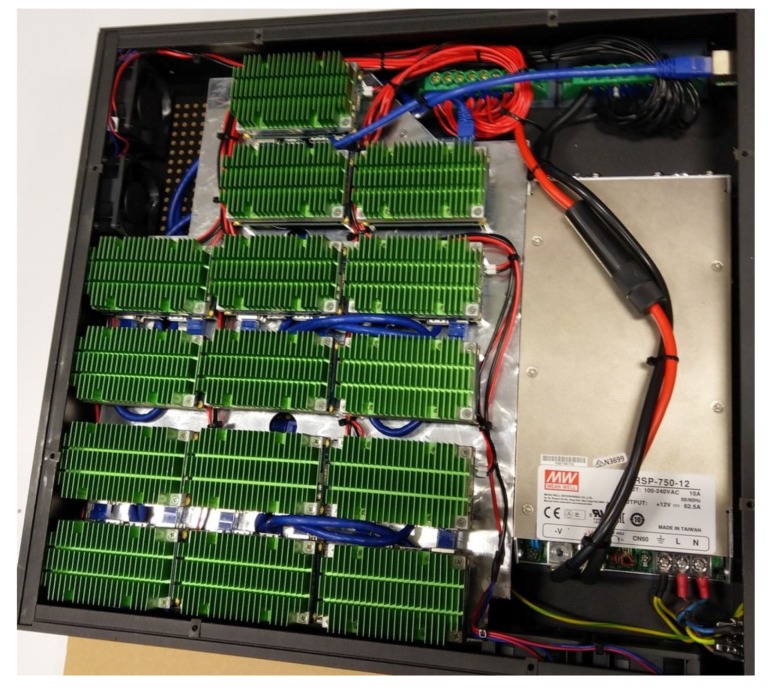
The 3U server version of the visual sensor hosting 15 NVIDIA Jetson TX2 units, each of these computing modules are able to process in real time one live video feed from a CCTV camera.

When LoRaWAN is not needed and the devices access the video feed of IP cameras (a typical situation for CCTV infrastructures), a 3U rack version has been designed and is shown in Figure 5. This rack unit contains 15 independent Jetson TX2 modules, each being able to process the feed of one camera. This setup has the advantage of resiliency: if one of the units in the rack fails, this does not impact the others and they can keep functioning.

Additional details about the detection and tracking steps are provided in the following subsections.

### 3.2. Detecting Objects: YOLO V3

Nowadays, many computer vision algorithms based on deep learning techniques are available to perform object detection in an image. In the context of traffic flow monitoring, it is important to select an algorithm that can perform the detection in real-time in an embedded system while maintaining a good level of accuracy. For those two reasons, YOLO V3 [37], a state-of-the-art and popular object detector based on fully convolutional deep neural network, is a good candidate. Compared to other algorithms, YOLO V3 offers a good equilibrium between speed and accuracy and can detect objects at three different scales. This last feature is also a critical requirement in our context as the observed size of a moving object depends on its distance from the camera.

Contrarily to previous existing methods, the YOLO (You Only Look Once) architecture runs an input image (scaled to a given input size) only once through the Darknet deep neural network (hence its name). The network is fully convolutional and contains 106 hidden layers gathered in residual blocks. It has been trained and adapted to detect the following six type of objects:

• pedestrian   • bus• bicycle   • truck• car   • motorbike

This network divides the image in three grids (one for each detection scale), where each cell of a grid predicts *K* bounding boxes. Each bounding box *B* is characterized by:

- its shape defined by the its centroid coordinates (x,y), its width *w* and height *h*;
- an object confidence score *O*; and
- six class probabilities $P_{i}$ (one for each object type).

If O<θ, where θ is a given confidence threshold, then *B* is discarded in order to remove the bounding boxes with the least confidence score. On the other hand, if O≥θ, then *B* is associated with the object type *o* such that $o=\arg\max_{i}P_{i}$.

**Figure 6:**
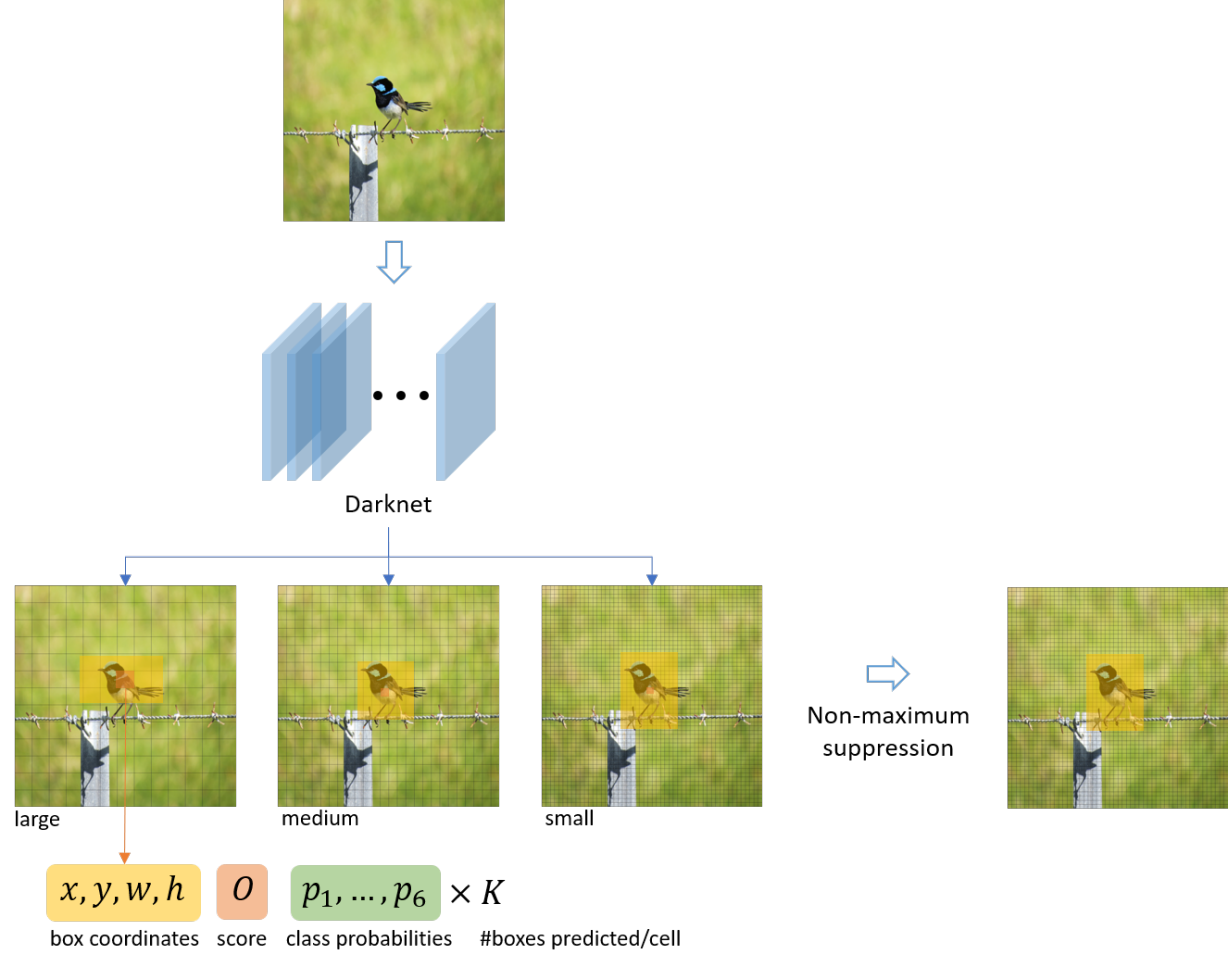
Architecture of YOLO V3 for object detection. A picture is passed through a fully convolutional neural network of 406 hidden layers in order to predict box coordinates and class probabilities at three different scales (large, medium, small). A non-maximum suppression algorithm is then applied to only retain the category and coordinates with the higher score.

Finally, to remove duplicate detection of the same object, the Non-Maximal Suppression (NMS) technique is applied. If we have an intersection-over-union (IoU) between two bounding boxes $B_{m}$ and $B_{m}$ greater than a predefined NMS threshold γ, i.e., if $IoU(B_{m},B_{n})\geq \gamma$, then the bounding box with the least objectness score is removed. The detection workflow is summarized in Figure 6.

For our application, YOLO V3 has been implemented in PyTorch 1.1 taking advantage of the CUDA cores available in the GPU of the NVIDIA Jetson TX2. The parameters’ values are listed in Table 1. The interested reader can find a fully detailed description of YOLO V3 and its performance in [37].

### 3.3. Tracking Objects: SORT

Once the detection task is done, the next step is to match the detected objects in the current frame with the ones from the previous frame. To perform this multiple object tracking (MOT) task, the Simple Online and Real-time Tracking (SORT) algorithm, fully detailed and benchmarked in [38], is used (A Python 3 implementation is freely available here: https://github.com/abewley/sort).

SORT has been developed with a major focus on efficiency in order to be used in real-time application, a critical requirement for our application, while still being offering excellent tracking performances. This is achieved by coupling two methods well known for their computational efficiency and their accuracy:

- a Kalman filter [39] to estimate the position of the bounding boxes in current frame from their previous locations; and
- the Hungarian algorithm [40] for optimally solving the problem of association between the predictions and the bounding boxes.

The state of each tracked object, or tracklet, *t* is modeled in the Kalman filter as follows:

$$t={\left[x,y,a,r,\dot{x},\dot{y},\dot{a}\right]}^{T}.$$

where

- *x* and *y* are the centroid coordinates of the object’s bounding box;
- *a* and *s* are the area and the aspect ratio of the object’s bounding box; and
- $\dot{k}$ is the velocity of the feature k∈{x,y,a}.

It can be noted that the aspect ratio is assumed constant. Only the geometric components of the bounding box computed by YOLO V3 are used to update *t*’s state, while the velocity components are determined by the Kalman filter. As the initial speed of *t* is unknown, the initial velocity components in the error covariance matrix used by the Kalman filter are set to larges values to reflect this uncertainty.

To pair detections with tracklets, the position and geometry of each tracklet’s bounding box are predicted by the Kalman filter in the current frame. The optimal assignment between the predictions and the detections is then performed by the Hungarian algorithm, where the cost matrix is given by the IoU between every detection-prediction pairs. According to the authors of SORT, using the IoU-based distance has the benefit of handling short-term occlusions [38]. After the assignment step, every association for which the IoU is lower than a threshold $IoU_{min}$ is rejected.

Two additional parameters, $hit_{min}$ and $age_{max}$, are also used to prevent the device’s memory to be completely filled, to mitigate the issue of false positive detection and to improve the tracking of objects that can be occluded for a maximum of $age_{max}$ frame. A tracklet is saved only if it has been seen at least in $hit_{min}$ frames and will be discarded if it has not been detected in $age_{max}$ frames in a row. Each new tracklet is given a unique identifier (Version 4-UUID).

The values of the tracking parameters used by the visual sensor are given in Table 2.

It should be noted that, when an object of interest leaves the field of view of camera and re-enters it later on, it will be associated with a new tracklet and a new id. The next section introduces the architecture used to collect the information from the network of sensors.

## 4. The Agnosticity Infrastructure

*Agnosticity* is the software framework developed for the Liverpool project. Its core idea is to rely on open source software and technologies as much as possible without making any assumptions on the type of sensors and communication protocols being used. The function of *Agnosticity* is to support the collection, storage and access of IoT data and to enable interoperability between different technologies thanks to the use of the OneM2M standard [41,42]. As the IoT quickly scales up, interoperability is becoming a crucial challenge to any Smart City application [43]. The multiplication of sensors, network protocols and usage delegates to the infrastructure level the capacity to ingest data from different sources and to provide a common access point. The Eclipse OM2M project is an open source implementation of oneM2M and SmartM2M standard. It provides a horizontal M2M service platform for developing services independently of the underlying network, with the aim to facilitate the deployment of vertical applications and heterogeneous devices. OM2M exposes a RESTful API providing primitive procedures for machine authentication, resource discovery, application registration, container management, synchronous and asynchronous communications, access rights authorization, groups organization, and re-targeting.

**Figure 7:**
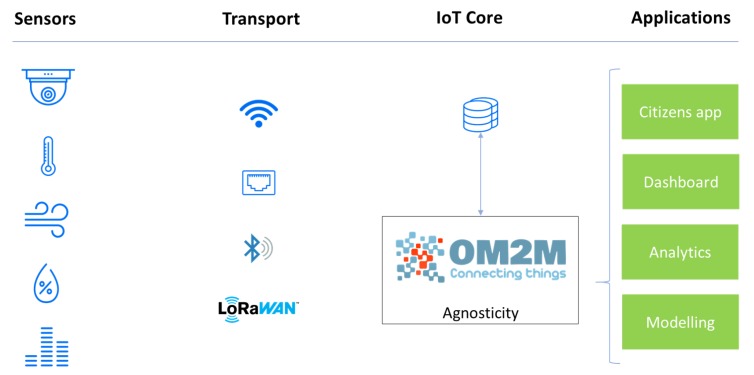
The general architecture of the project. The *Agnosticity* software stack relies on well established open-source software. The data collection and access is ensured by the open-source implementation of the OM2M standard.

Figure 7 introduces the general architecture of the Agnosticity framework implemented in this project. Fifteen fixed visual sensors are connected to the CCTV network and push data to the OM2M platform through HTTP Post. Five Mobile visual sensors and 20 air quality sensors rely on LoRaWAN and The Things Network to publish data. A specific plugin automatically recovers the data from The Things Network using the MQTT broker and re-publishes the data to the OM2M platform through HTTP Post. Thus, all data are directly available on the OM2M platform under a specific container. A subscription mechanism allows it to automatically store each into a dedicated database for long-term storage.

**Figure 8:**
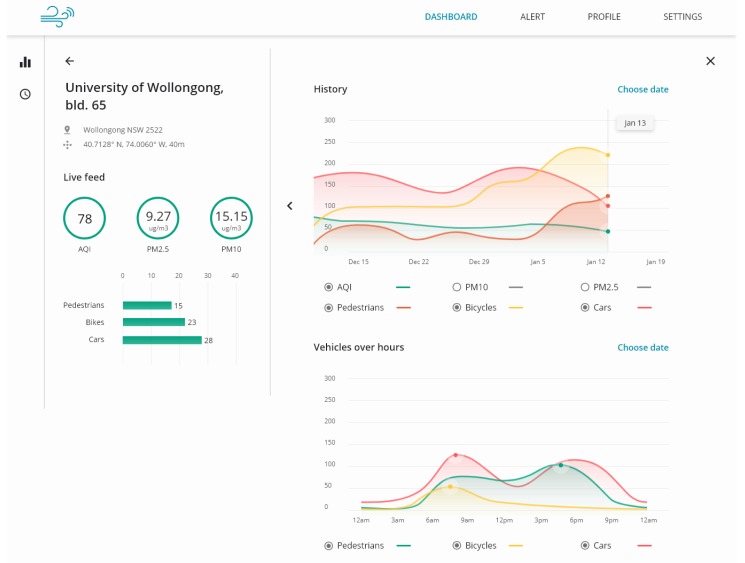
The web-based interactive dashboard used to represent the collected data from the different sensors deployed for the Liverpool project. The interface is responsive and can be used both on desktop and mobile browsers.

Different applications can be built on top of the Agnosticity framework, by either directly accessing data using the OM2M RESTful API, or by requesting data from the dedicated database. For example, the web-based dashboard shown in Figure 8 has been designed to visually explore the collected data from the different sensors.

Now that the sensor and the Agnosticity architecture have been introduced, validation experiments are illustrated in the following section.

## 5. Validation Experiments

We investigated the performance of the sensor in terms of speed, accuracy and system’s utilization. We first evaluated the accuracy and performance of the sensor to detect pedestrians on a validation dataset extracted from the literature. This was followed by an analysis of the system’s and network’s utilization during from a real world experiment where the sensor was connected to a CCTV.

### 5.1. Accuracy and Performance

We evaluated the accuracy and performance of the sensor on the Oxford Town Center Dataset [28]. The video gives a high definition view (1920 × 1080 @ 25 fps) of a busy town center street from a CCTV perspective. The validation experiment was done on the first 4500 frames of the video corresponding to 3 min. In this sequence, 230 pedestrians and their position were annotated in the video by the authors of the dataset. Therefore, we assessed the performance of our sensor by comparing its results to the ground truth.

Table 3 summarizes the performance results with basic statistics computed over the 4500 frames for the following variables:

- detection: the number of objects detected by the sensor;
- true: the number of object annotated in the dataset, i.e., the ground truth;
- error: the difference between detection and true;
- |relativeerror|: the relative error computed as:
  $$\frac{\left|error\right|}{true};$$
- accuracy: the accuracy defined by:
  $$\frac{detection}{true};$$
  and
- fps: the inverse of the time required to process a frame of the video, i.e., the number of frames per second (FPS) processed by the sensor.

We now dive deeper into the validation analysis.

**Figure 9:**
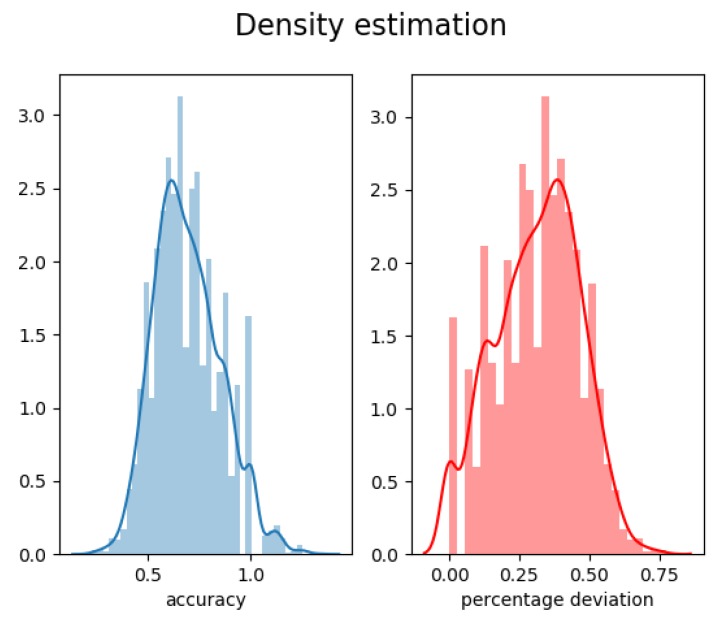
Kernel density estimation of the accuracy (**left**) and percentage deviation (**right**) computed across the 4500 frames of the Oxford dataset.

Those initial results demonstrate that the sensor had a mean accuracy of 69% with a median relative error of 33%. The error also indicated that the sensor tended to underestimate the number of detection, impacting the relative error and the accuracy. The distribution of the accuracy and the relative error across all the frame is given in Figure 9.

**Figure 10:**
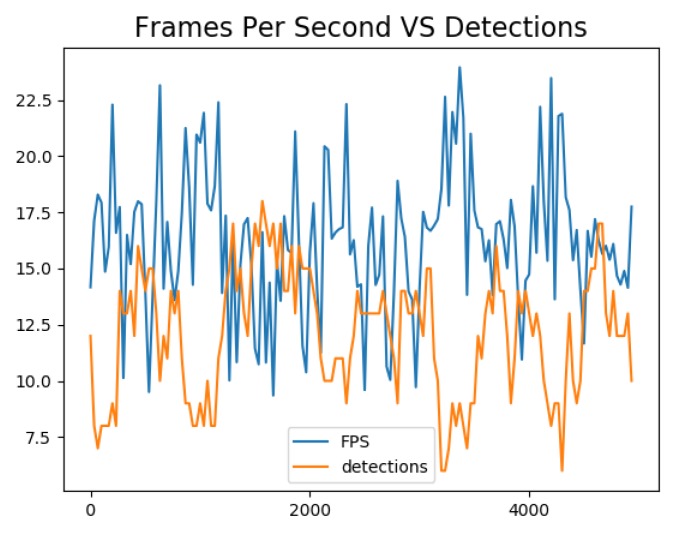
Evolution of the number of frame per seconds processed (FPS) by sensor (blue) and the number of detected objects (red) over time. It can be seen that the FPS are higher when the number of detection is lower. The drop in FPS is mainly due to the SORT algorithm as there are more objects to be tracked, a task not taking advantage of the CUDA cores available on the Jetson TX2.

Figure 10 shows the evolution of the number of FPS and the number of detected pedestrians over time. We can see that the two curves were anti-correlated. Indeed, a higher number of detection generally led to a decrease in the FPS performance. This was due to the implementation of the SORT tracking algorithm not taking advantage of the CUDA cores available on the Jetson TX2 and acting therefore as a bottleneck. Future iteration of the algorithm should address this limitation by implementing this tracker on the GPU.

**Figure 11:**
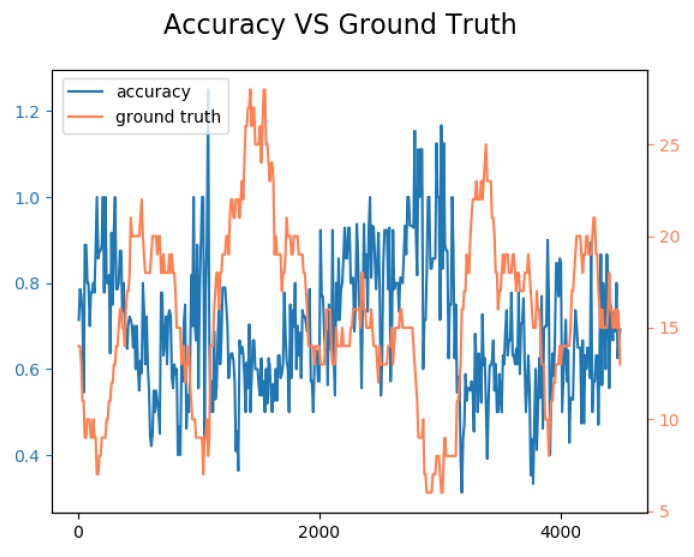
Evolution of the accuracy (blue lines, ideal is 1.0) in accordance with the ground truth (orange lines) over time. Accuracy is better with small groups and decreases with large crowds.

We illustrate in Figure 11 the evolution of the accuracy of the sensor and the ground truth for over the 4500 frames. It can be seen that the curves were anti-correlated: the accuracy was higher when the number of ground truth detection was low, and the accuracy decreased with large crowds. This can be explained by occlusions that can occur in large crowds. Indeed, if one person is hidden by another one, the algorithm might only detect one of the two. This is partly due to the behavior of YOLO V3, which applies a non-maximum suppression algorithm to situations where multiple bounding box are overlapping.

**Figure 12:**
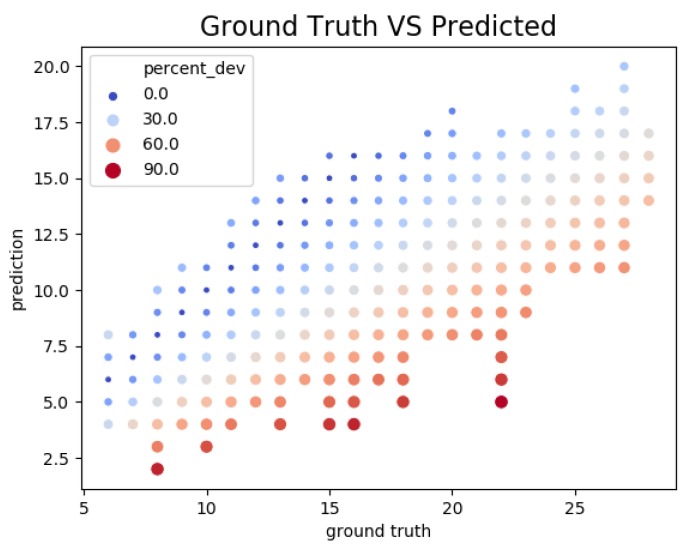
Number of ground truth detection against the number of detection. We observe that the relationship is linear, meaning that the algorithm manages to capture trends.

Figure 12 is a scatter plot of the number of ground truth detection against the number of detection made by the sensor. It can be observed that the relationship was linear, i.e., the larger was the ground truth, the greater was the number of object detected by the sensor. This figure also confirms that the sensor was more prone to false negative errors than false positives, as it was underestimating the number of detections. While being a source of error, underestimation is less problematic than overestimation in our context of traffic monitoring. A more thorough investigation of the error rate would definitely benefit to the accuracy of our algorithm. Nonetheless, the trends in terms of number of detected objects is correct, resulting in overall satisfactory performance and accuracy for the use cases of this sensor.

### 5.2. System and Network Utilization

**Figure 13:**
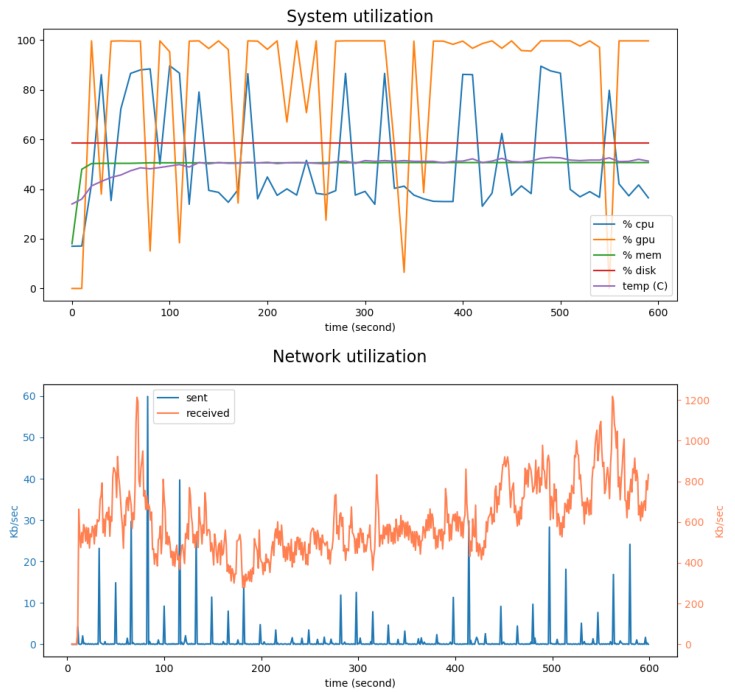
A 15 min monitoring of the CPU, GPU, memory and disk usages, average temperature (**top**) and network utilization (**bottom**) during a real world deployment of the sensor. Over this period, 280 unique objects have been detected.

Figure 13 shows the evolution of CPU, GPU, memory and disk usages, average temperature and network utilization every 2 s during a real world deployment of the sensor. During this 10-min experimentation, the sensor was connected to a CCTV monitoring the entrance of a building and the street nearby.

It can be seen in the top panel that the disk and memory usage, and the temperature of the system were stable. The GPU was occasionally underutilized at some points during this experiment, dropping from 100% to less than 40%. As mentioned above, this can be explained by the fact that the tracking algorithm was implemented on the CPU, and acted as a bottleneck. Indeed, drops in GPU usage were related to peaks in CPU usage.

The bottom panel displays the inbound and outbound network bandwidth used every seconds. The incoming data corresponded to the acquisition of a frame from the CCTV. The transmitted data corresponded to the publication of data to the Agnosticity platform. One outgoing peak is visible every 1 min, corresponding the sending rate of the sensor. We can easily see that there were fewer outgoing data than incoming dat. This was because only meta-data extracted from the frames such as counts and trajectories were transmitted. Consequently, not a lot of bandwidth is required by the sensor.

The next section introduces two real world applications.

## 6. Applications

This section details two real-world applications of the visual sensor. The first one consisted of a 1 h monitoring of foot traffic flow within a building during an emergency evacuation. The second introduced results from a one-week traffic monitoring exercise in the city of Liverpool. Those two applications demonstrated the validity of the approach in real-life situations. In both applications, the CCTV cameras were streaming a full high-definition video feed at 25 frame per seconds.

### 6.1. Indoor Deployment

As an initial experiment, the visual sensor was deployed inside a building to monitor the flow of people going through the first floor of the SMART Infrastructure Facility building of the University of Wollongong. This building houses different labs and teaching activities, and, thus, is a good place to monitor pedestrian activity. The camera was put in front of a stairway that is the main access to the first floor.

**Figure 14:**
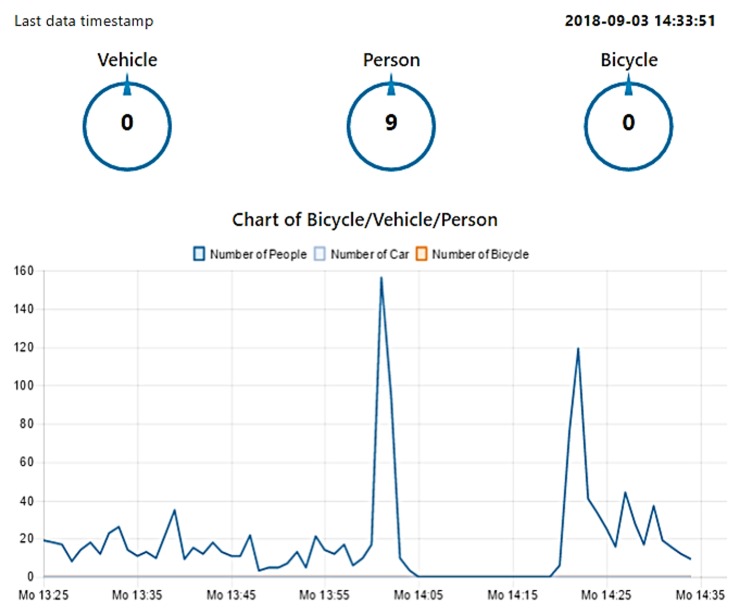
Plot of the number of people detected inside a building over one hour. The two peaks correspond to the start and end of the fire alarm event.

Figure 14 illustrates the evolution of the number of people detected by the sensor, during the experiment that took place between 14:30 and 15:30 on a regular working day. It can be seen that no vehicle or bicycle was detected, which is to be expected inside a building. Two peaks could also be observed, without any detection made in between. This can be explained by the fire alarm that went off during the experiment. Fortunately, it was a false alarm, which was not caused by the authors of this paper to gather more data. Interestingly, no one was detected during the fire alarm event, meaning that the building was effectively evacuated. One can also observe that the second peak was lower than the first one, indicating that less people returned after the firemen deemed it was safe to do so. These first findings suggest that this sensor could be well suited to detect abnormal crowd movements.

**Figure 15:**
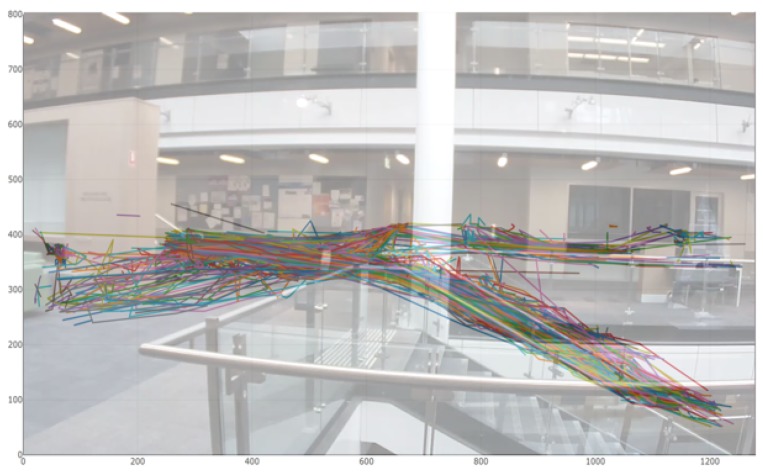
Trajectories followed by the individuals detected and tracked by the sensor. Each of the 631 lines represent one individual.

**Figure 16:**
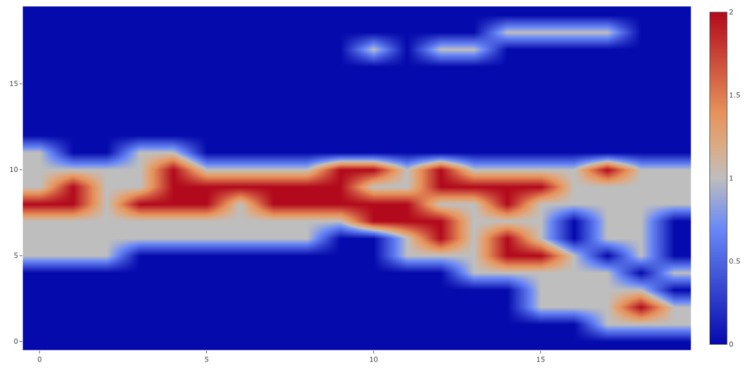
Heat map of the maximum number of individual detected in the field of view of the sensor.

During this 1 h experiment, 631 people were detected and tracked. The resulting trajectories are shown in Figure 15 overlaid to the actual field of view of the sensor. It can be seen that the trajectories corresponded to what one would have expected (no trajectories in between floors for instance). In addition to those trajectories, we could also derive other interesting information from the collected data, for instance a heat map such as the one in Figure 16 highlighting the maximum of number of detection in the field of view of the sensor during the experiment. Other metrics could include the maximum or minimum of time spent at a specific location, the relative speed of the detected boxes or the automatic detection of anomalous behaviors such as unauthorized line crossing.

### 6.2. Outdoor Deployment: Liverpool

**Figure 17:**
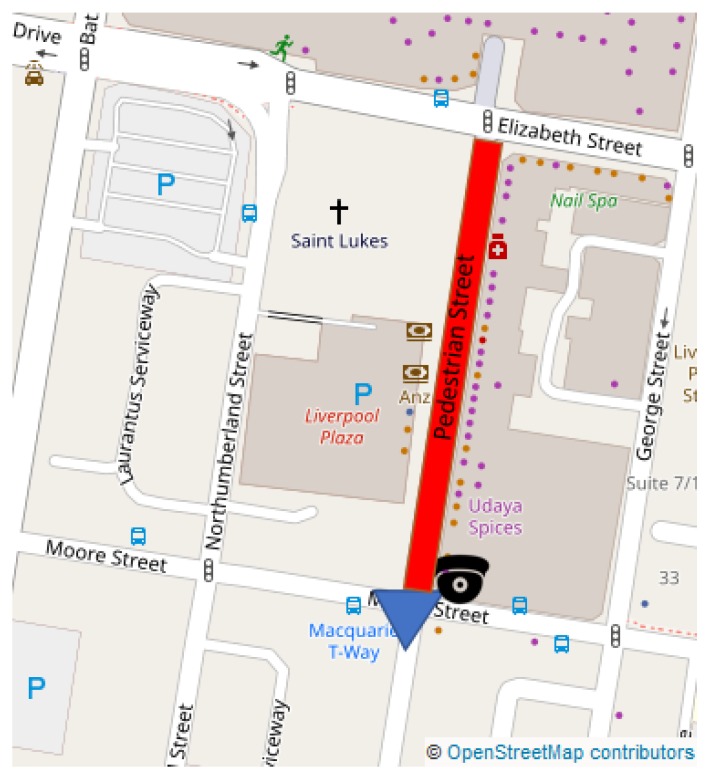
The sensor is located at the center of the city, next to a pedestrian street (highlighted in red). The field of view of the camera is represented in blue.

Following the initial tests of the sensor in a controlled environment, 20 visual sensors have been deployed in the center of Liverpool (Figure 1). This application will focus on the camera whose location is illustrated in Figure 17. This location is next to a pedestrian street with a huge pedestrian activity due to the proximity of a lot of shops and restaurants. The camera is overlooking an intersection with three crosswalks.

**Figure 18:**
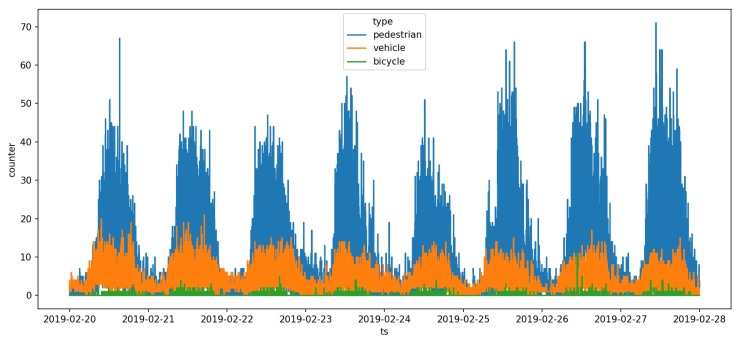
Number of pedestrian, vehicle and bicycle detected by the sensor between 20 February 2019 and 27 February 2019. Each data point represents the number of objects of a specific type detected over the last minute.

**Figure 19:**
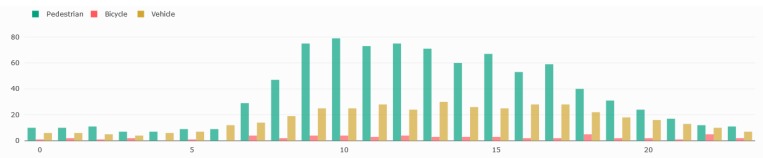
Number of detection pedestrians (green), bicycles (red), and vehicles (yellow) detected hourly on 23 February 2019.

Figure 18 shows the counting results from one week during the period going from 20 February 2019 to 27 February 2019. It displays the number of pedestrians, vehicles and bicycles seen every minute during those eight days. In this figure, we can easily see the daily circadian rhythm of the city, where most of the activity was between 08:00 and 16:00. This is better seen in Figure 19 illustrating the number of hourly detection.

The last three days seemed to have a higher pedestrian activity than the other days. Day 5 (24 February 2019) had the lowest pedestrian activity and corresponded to a Sunday. Each day, the peak activity was around noon. If we look at the vehicle graph (in orange), we can see two daily peaks of activities, the first one around 09:00 and the second one around 16:00. Those results seem to correspond with what we can expect from such an intersection but have to be properly analyzed by urban planners. Nonetheless, it illustrates the capacity of the sensor to detect variations in the daily circadian rhythm of traffic flows.

**Figure 20:**
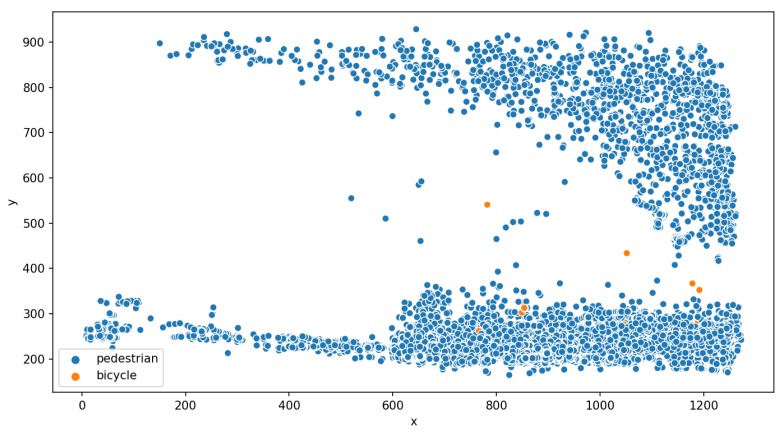
Plot of the pixel coordinates (*X*,*Y*) of the detected pedestrians (blue) or bicycles (orange) in the frame for the day 23 February 2019. Each dot represents the centroid of the bounding box corresponding to an item that has been detected at those coordinates.

Figure 20 displays the coordinates of every detection of pedestrians and bicycles during the day of 23 February 2019. During that day, 20,399 unique objects were detected. Each blue dot corresponds to the detection of a pedestrian and each orange dot corresponds to the detection of a bicycle. One dot corresponds to one detection, but one individual was typically detected multiple times while being in the field of view of the camera. This visualization allows consideration of the spatial distribution of the detection over the frame. In the current context, it allows considering the pedestrian flows crossing the street at a crosswalk (corresponding to the top and bottom of the graph). Some of the pedestrians were detected outside of the crosswalk path. The detection of bicycles indicated a mix of traffic between pedestrians and cyclists. However, the number of detected bicycles was extremely low (which is in accordance with what citizens has expressed during the community workshop).

**Figure 21:**
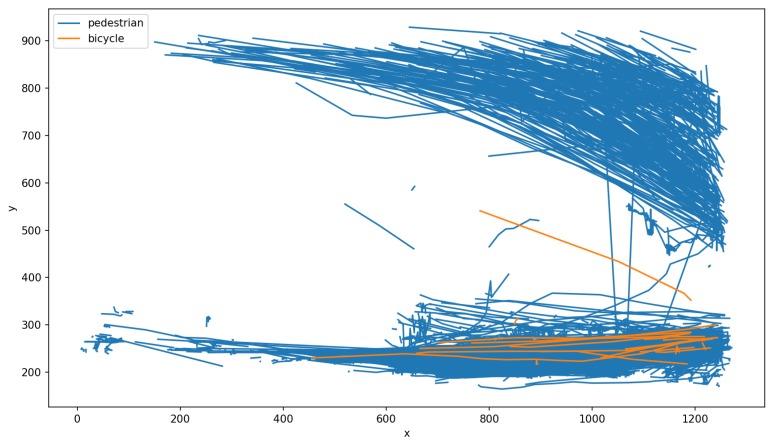
Trajectories of pedestrians and bicycles within the frame during the day of 23 February 2019. Two flows of pedestrians are visible.

The resulting tracks followed by the pedestrians and cyclist are shown in Figure 21. Two different flows are visible (one from the top left to the middle right, the other from the bottom left to the bottom right). These two flows reveal the mobility patterns on this intersection.

The different results shown in this section aim to highlight the data made available to the urban planners. An extended analysis of those data by urban planners is ongoing.

## 7. Conclusions and Future Work

This paper details an original visual sensor, part of an IoT solution for monitoring the traffic flow of bicycles, vehicles and pedestrians. The complete solution proposes to deploy more visual sensors, and to collect data through the Agnosticity framework. The main objective of this framework, also introduced in this work, is to enable interoperability by using the OM2M open source software, implementing the oneM2M standard.

Since its hardware is based on the NVIDIA Jetson TX2 embedded computing platform and all computation is made onboard, the sensor is an edge-computing device. Its software couples YOLO V3, a popular convolutional deep neural network, with SORT, a real-time tracking algorithm. Meta-data are then extracted and transmitted using either Ethernet or LoRaWAN protocols. As the solution relies on open-source software, it is extensible, offers a high level of maintainability and can be easily replicated.

As only meta-data are transmitted, and no raw or processed images, the sensor offers a privacy compliant tracking solution. This also means that the sensor can be paired with the existing CCTV infrastructure typically owned by city councils, thus optimizing their use and adding new value to the network as it is now possible to exploit the vast amount of collected video data. In addition, thanks to the long-range LoRaWAN radio protocol, additional camera can be easily deployed in the field where no conventional Internet connectivity is available.

The use of the sensor in two different environments (indoor and outdoor) has shown that the proposed approach is promising and is able to detect different types of traffic flows and trends. The solution was tested in the city of Liverpool (NSW, Australia), with 20 sensors deployed. This allows the city council to monitor in real-time the traffic conditions, as well as to explore the traffic flows through a dashboard. An extended analysis of the collected data by urban planners over a longer period of time will assess how these data might be used daily to better plan mobility within the city.

Future work includes the improvement of the detection and tracking algorithms performance and accuracy. For instance, being able to quantify the error made by the detection algorithm can help improve and correct the counts of detected object. Using the NVIDIA Xavier platform instead of the Jetson TX2 is a good candidate for improving the performance. Preliminary experiences suggests that, thanks to the double number of CUDA cores compared to the TX2, the Xavier is able to perform the detection and tracking twice as quickly.

Another area of evolution is the use of a production-oriented deep learning framework. The current implementation relies on Pytorch 1.0, which is possibly a performance bottleneck as it mainly focuses on the ease of prototyping, even though the latest version introduces major improvements in terms of computational optimization. Porting YOLO V3 to other frameworks such as Caffe, Tensorflow paired with TensorRT to evaluate their impact on the overall performance is likely to have a positive impact on the performances. Finally, implementing the SORT algorithm (or any other state-of-the art real-time MOT) such that it also takes advantage of GPU computing will be investigated.

## Figures and Tables

**Table 1:** YOLO V3 parameters for the detection task.

Parameter	Value
Input size	416 × 416 pixels
Small scale detection grid	52 × 52 cells
Medium scale detection grid	26 × 26 cells
Large scale detection grid	13 × 13 cells
Number of bounding box per cell *K*	3
Confidence θ	0.9
NMS γ	0.5

**Table 2:** SORT parameters for the tracking task.

Parameter	Value
Minimum hits $hit_{min}$	3
Maximum age $age_{max}$	40
Threshold $IoU_{min}$	0.3

**Table 3:** Summary of the performance results with basic statistics computed over the 4500 frames. Those results demonstrate that the algorithm under estimate the number of detection, but manages to have a good speed (fps).

	Detection	True	Error	Relative Error	Accuracy	fps
**mean**	10.52	15.87	−5.34	0.31	0.69	19.57
**standard deviation**	2.80	4.69	3.35	0.15	0.15	3.49
**minimum**	2.00	6.00	17.00	0.00	0.22	4.63
**25th-percentile**	8.00	13.00	−8.00	0.21	0.57	17.28
**median**	11.00	16.00	−5.00	0.33	0.66	19.77
**75th-percentile**	13.00	19.00	−3.00	0.42	0.78	22.22
**maximum**	20.00	28.00	2.00	0.77	1.33	22.99

## Appendix A. Technical Specifications

The main technical specifications of the NVIDIA Jetson TX2 and Pycom LoPy 4 modules are detailed in Table A1 and Table A2, respectively.

**Table A1:** Specification of the NVIDIA Jetson TX2.

Feature	Detail
CPU	ARM Cortex-A57 (quad-core) @ 2 GHz + NVIDIA Denver2 (dual-core) @ 2 GHz
GPU	256-core Pascal @ 1300 MHz
Memory	8 GB 128-bit LPDDR4 @ 1866 Mhz | 59.7 GB/s
Storage	32 GB eMMC 5.1, SDIO, SATA
Decoder	4 K × 2 K 60 Hz Decode (12-Bit Support)
Supported video codecs	H.264, H.265, VP8, VP9
Wireless	802.11a/b/g/n/ac 2×2 867 Mbps, Bluetooth 4.1
Ethernet	10/100/1000 BASE-T Ethernet
USB	USB 3.0 + USB 2.0
PCie	Gen 2 | 1 × 4 + 1 × 1 or 2 × 1 + 1 × 2
Miscellaneous I/O	UART, SPI, I2C, I2S, GPIOs, CAN
Socket	400-pin Samtec board-to-board connector, 50 × 87 mm
Thermals	−25 °C to 80 °C
Power	15 W, 12 V

**Table A2:** Specifications of the Pycom LoPy 4.

Feature	Detail
CPU	Xtensa^®^ 32-bit (dual-core) LX6 microprocessor, up to 600 DMIPS
Memory	RAM: 520 KB + 4 MB, External flash: 8 MB
Wireless	Wifi 802.11b/g/n 16 Mbps, Bluetooth BLE, 868/915 MHz LoRa and Sigfox
Lora and Sigfox connectivity	Semtech SX1276
Miscellaneous I/O	GPIO, ADC, DAC, SPI, UART, PWM
Thermals	−40 °C to 85 °C
Power	0.35 W, 3.3 V
